# Structural Characterization Study of a Lipid Nanocapsule Formulation Intended for Drug Delivery Applications Using Small-Angle Scattering Techniques

**DOI:** 10.1021/acs.molpharmaceut.1c00648

**Published:** 2022-02-28

**Authors:** Dileep Urimi, Maja Hellsing, Najet Mahmoudi, Christopher Söderberg, Ronja Widenbring, Lars Gedda, Katarina Edwards, Thorsteinn Loftsson, Nicolaas Schipper

**Affiliations:** † RISE Research Institutes of Sweden, Division Bioeconomy and Health, Chemical Process and Pharmaceutical Development, Forskargatan 18, Södertälje 151 36, Sweden; ‡ Faculty of Pharmaceutical Sciences, School of Health Sciences, University of Iceland, Hofsvallagata 53, Reykjavík IS-107, Iceland; § ISIS Pulsed Neutron and Muon Source, Rutherford Appleton Laboratory, Didcot OX11 0QX, U.K.; ∥ Department of Chemistry − Ångström laboratory, Uppsala University, Box 573, Uppsala SE-751 23, Sweden

**Keywords:** lipid nanocapsules, LNC, nanoparticles, core−shell structure, DF003, small-angle X-ray scattering, SAXS, small-angle neutron scattering, SANS

## Abstract

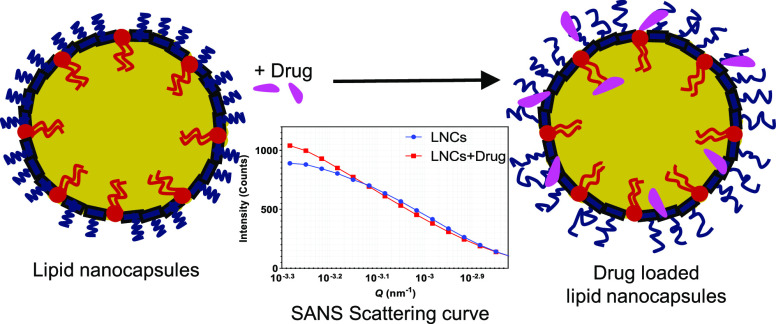

Lipid nanocapsules (LNCs) are increasingly being used for various drug delivery applications due to their versatile nature and ability to carry a wide variety of therapeutic drug molecules. In the present investigation, small-angle X-ray (SAXS) and neutron scattering (SANS) techniques were used to elucidate the structure of LNCs. Overall, size measurements obtained from SAXS and SANS techniques were complemented with dynamic light scattering, zeta potential, and cryogenic transmission electron microscopy measurements. The structural aspects of LNCs can be affected by drug loading and the properties of the drug. Here, the impact of drug loading on the overall structure was evaluated using DF003 as a model drug molecule. LNCs with varying compositions were prepared using a phase inversion method. Combined analysis of SAXS and SANS measurements indicated the presence of a core–shell structure in the LNCs. Further, the drug loading did not alter the overall core–shell structure of the LNCs. SANS data revealed that the core size remained unchanged with a radius of 20.0 ± 0.9 nm for unloaded LNCs and 20.2 ± 0.6 nm for drug-loaded LNCs. Furthermore, interestingly, the shell becomes thicker in an order of ∼1 nm in presence of the drug compared to the shell thickness of unloaded LNCs as demonstrated by SAXS data. This can be correlated with the strong association of hydrophilic DF003 with Kolliphor HS 15, a polyethylene glycol-based surfactant that predominantly makes up the shell, resulting in a drug-rich hydrated shell.

## 1 Introduction

Lipid nanocapsules (LNCs) are a versatile carrier system often used in drug delivery applications. LNCs are prepared by a phase inversion method^1^ where the formation of LNCs is governed by a temperature-dependent behavior of a hydrophilic surfactant such as Kolliphor HS 15 (polyethylene glycol (15)-hydroxystearate). The properties of LNCs can be varied greatly by changing their composition, and they can be prepared in a variety of sizes to suit different applications. LNCs also offer a possibility to load both hydrophilic and hydrophobic drug molecules.^2^ Due to their biomimetic nature,^3^ LNCs have previously been studied for their suitability as drug delivery systems for treating conditions like cancer, antibiotic resistance, and ocular conditions, including age-related macular degeneration.^4−9^

LNCs comprise a hydrophobic oily core that is surrounded and stabilized by a combination of PEGylated surfactants and phospholipids.^3,10,11^ Characterization tools such as dynamic light scattering, cryogenic transmission electron microscopy (cryo-TEM), drop tensiometry, Langmuir balance, and atomic force microscopy have been used to understand the structural properties of LNCs.^3,11,12^ Previous studies demonstrate a preferential orientation of phospholipids toward the oily core, whereas a PEGylated surfactant orients toward the aqueous phase, collectively forming a tensioactive cohesive membrane surrounding the oily core.^10,11^ Direct confirmatory studies using scattering techniques will provide further information on the internal structure of LNCs. Understanding the structural aspects of LNCs will give more insight into what factors govern their formation and the mechanism of drug interaction, and enable optimization of drug delivery systems for various therapeutic applications.^11,13,14^ Furthermore, the structural information provides additional insight about the mechanisms of LNC interactions with cell membranes. Drugs may selectively deposit in certain parts of LNCs, or they may distribute uniformly depending on their physicochemical properties. By studying the structural aspects of LNCs in combination with understanding the drug localization, a better correlation of in vitro and in vivo performance can be made. This will in turn allow for a further improvement in the nanoparticle properties and performance to suit diverse applications.

Loading of drugs into nanoparticles may induce structural changes of the nanoparticles that have the potential to affect the drug loading efficiency. This is advantageous in certain cases; for instance, encapsulation of doxorubicin into liposomes may result in nanoprecipitation inside the core of liposomes. As a result, morphologically they look like ellipsoidal vesicles compared to spherical structures when they have no drug.^15,16^ This nanoprecipitation ultimately results in increased drug loading and a prolonged drug release.^17^ However, many novel drug delivery systems that have showed potential for treating various diseases remain poorly characterized at the nanoscale, and how the drug interacts with the drug delivery systems is still unknown in many cases.^18,19^ Structural elucidation will thus offer possibilities for a better formulation optimization to suit the desired applications.

A diverse range of experimental methods and tools are available for a detailed structural characterization of nanoparticles. The selection of suitable techniques depends on the part of the system to be studied and on the type and composition of nanoparticles and drugs. More general characterization techniques like dynamic light scattering (DLS) and cryo-TEM are very useful tools, but they may not give a complete and necessary information about the structure of the formulation. DLS, for example, gives information about the apparent hydrodynamic particle size and polydispersity, based on the estimated diffusion coefficient, but it does not measure the internal structure of the particles. However, DLS is often easily accessible and does, in most cases, not require specific sample modifications. On the other hand, advanced characterization techniques like tomography and small-angle X-ray and neutron scattering (SAXS and SANS) allow more accurate size estimation as well as provide insight into the internal structure of nanoparticles.^14^ By combining complementary techniques, it is possible to fully characterize particle size, size distribution, shape, internal structure, and intermolecular interactions, resulting in better understanding and prediction of the formulation properties.^20^

In this investigation, in addition to studying unloaded LNCs, drug-loaded LNCs were prepared with DF003, a novel cyclic guanosine-3′,5′-monophosphate (cGMP) analogue. In previous studies, DF003 was found to provide a protective effect on the survival of photoreceptors in cell cultures and animal models of retinal degeneration.^21^ The present investigation combines different characterization techniques to unravel the structural properties of unloaded and drug-loaded LNCs. SAXS and SANS are mainly used to determine the structure of LNCs and any influence of drug loading or temperature on the structure. Additionally, an attempt to understand the preferential distribution of drug in LNCs is made. Observations from these experiments are compared and complemented with DLS, zeta potential, and cryo-TEM studies.

## 2 Theory of Small-Angle Scattering

SAXS and SANS are two small-angle scattering techniques, which offer detailed investigation possibilities of samples at colloidal length scales. The structure of particles is obtained from the absolute scattering intensity of X-rays and neutrons, which can be expressed as eq 1.^22,23^
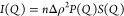
1where *n* is the number density of the particles in the sample, and ρ is the scattering length density (SLD) difference between the particles and the dispersion medium (can be calculated from known values of the measured systems).^24,25^*Q* represents the magnitude of the scattering wave vector *Q* = 4π/λsin(θ/2), where θ is the scattering angle and λ is the wavelength of the incident beam. *P*(*Q*) represents the form factor, which is related to the size and shape of the nanoparticles in the dispersion medium, and *S*(*Q*) represents the structure factor and is related to the interparticle interactions in the dispersion medium.

SAXS can detect nanoscale density differences of electrons in a sample. This means that it can resolve nanoparticle size distributions, study the size and shape of (monodisperse) macromolecules, determine pore sizes, study characteristic distances of partially ordered materials, and much more. LNCs are soft nanoparticles with similar electron density profiles across their whole internal structure, and SAXS alone may not be enough to study their internal structural layers. SANS is in many respects very similar to SAXS, but SANS has a higher sensitivity to lighter elements and has the possibility of isotope labeling. In X-ray scattering, photons interact with the electronic cloud, so the larger the element, the larger the effect. In neutron scattering, neutrons interact with nuclei of atoms, and the interaction is therefore sensitive to isotopes; some light elements like deuterium show similar scattering cross sections as heavy elements. In aqueous dispersions, combinations of normal (H_2_O) and heavy (D_2_O) water can be used to achieve good signal from a sample. Additionally, by matching the contrast of part(s) of the particle with the dispersion medium, signal can be obtained from a specific region in the particles of interest, e.g., shell of core–shell nanoparticles. Hence, with the proper contrast differences, one can study individual components in complex structures as for example nanoparticles like LNCs. By preparing LNCs with deuterated components, it is possible to extract detailed information about the internal structure of LNCs.^26^ SANS with contrast variation provides information about the average particle size, shape, internal structure, and interactions between particles.

## 3 Experimental Section

### 3.1 Materials

Labrafac lipophile WL 1349, (caprylic–capric acid triglycerides at 54.0%:45.2% as per the certificate of analysis, Ph. Eur. Grade, Gattefossé, France), Kolliphor HS 15 (polyethylene glycol (15)-hydroxystearate, Ph. Eur. Grade, BASF), Phospholipon 90 H (hydrogenated phosphatidylcholine ≥90%, Lipoid, Germany), octanoic-d15 acid, decanoic-d19 acid, sodium chloride, and 50 kD Amicon Ultra-0.5 Centrifugal Filter Units (Sigma-Aldrich Sweden AB, Stockholm) were used in preparation and characterization of LNCs. Drug, DF003 (β-phenyl-1,*N*^2^-etheno-8-bromoguanosine-3′,5′-cyclic monophosphorothiotic acid, sometimes referred to as CN03 in previous publications), was synthesized within RISE Research Institutes of Sweden (Södertälje, Sweden) as part of the *trans*Med project (H2020-MSCA-765441) to prepare drug-loaded LNCs. Milli-Q water from ELGA, Purelab Prima and D_2_O (Cambridge Isotope Laboratories, Inc.) were utilized for all experiments. All other excipients and reagents were of analytical grade. The chemical structures of Kolliphor HS 15 and DF003 are provided in the Supporting Information (Figures SI1 and SI2).

## 4 Methods

#### 4.1.1 Preparation of LNCs

A phase inversion method was employed for preparing LNCs as described by Valcourt et al.^1^ and Urimi et al.^9^ In short, for preparing fully hydrogenated LNCs (h-LNCs), 620 mg of Labrafac, 480 mg of Kolliphor HS 15, 50 mg of sodium chloride, 40 mg of Phospholipon 90 H, and 1.7 mL of purified water/deuterium oxide (D_2_O) were heated to 50 °C until a clear solution was observed. This clear solution was then further heated to 90 °C followed by cooling it to 60 °C. Three such heat–cool cycles were applied followed by rapid addition of 7.1 mL of cold water or D_2_O near the phase inversion temperature during the last heat–cool cycle. This resulted in spontaneous formation of LNCs. Additionally, LNCs with a deuterated core (d-LNCs) were prepared with a combination of Labrafac and a mixture of deuterated fatty acids as an oil phase to achieve higher contrast in the core, to be suitable for SANS experiments. For this, 95% of Labrafac was mixed with a 5% mixture of octanoic-d15 (dC8) and decanoic-d19 acid (dC10) (dC8/dC10 corresponds to 54.0%:45.2% as per the certificate of analysis of Labrafac). Using this as an oil phase, d-LNCs were prepared in a similar manner as described above. All samples were filtered using a 0.22 μM membrane filter before further characterization. The composition of h-LNCs and d-LNCs is given in Table 1.

**Table 1:** Composition of Hydrogenated (h-LNCs) and Deuterated (d-LNCs) LNCs

excipient	composition of h-LNCs (% w/v)	composition of d-LNCs (% w/v)
Labrafac lipophile WL 1349	6.2	5.89
octanoic-d15 acid (dC8)		0.17
decanoic-d19 acid (dC10)		0.14
Kolliphor HS 15	4.8	4.8
Phospholipon 90 H	0.4	0.4
sodium chloride	0.5	0.5
Milli-Q water/deuterium oxide (D_2_O)	q.s.	q.s.
DF003^a^	0.19	0.19

a DF003 is only present in drug-loaded LNCs.

#### 4.1.2 Particle Size and Zeta Potential Measurements

The mean particle size and polydispersity index (PDI) of unloaded and DF003-loaded LNCs were measured using a Malvern Zetasizer Nano ZS setup (Malvern Instruments, U.K.) at a backscattering detection angle of 173°. Using the same setup, the zeta potential of the LNCs was determined by measuring the electrophoretic mobility of the samples in a folded capillary cell and then applying the Smoluchowski equation. The samples were measured in the concentration range of 1.2–62 mg/mL after making necessary dilutions of the original formulation.

#### 4.1.3 Morphology by Cryogenic Transmission Electron Microscopy

Drug-loaded LNCs with and without a deuterated core were analyzed by cryo-TEM as described by Almgren et al.^27^ Samples were equilibrated at 25 °C and at a high relative humidity within a climate chamber. A small drop of sample was deposited on a carbon-sputtered copper grid precovered with a perforated polymer film. Excess liquid was thereafter removed by blotting with a filter paper, leaving a thin film of the solution on the grid. The sample was vitrified in liquid ethane and transferred to the microscope, continuously kept below −160 °C, and protected against atmospheric conditions. Analyses were performed with a Zeiss Libra 120 transmission electron microscope (Carl Zeiss AG, Oberkochen, Germany) operating at 80 kV and in zero-loss bright-field mode. Digital images were recorded under low-dose conditions with a BioVision Pro-SM Slow Scan CCD camera (Proscan elektronische Systeme GmbH, Scheuring, Germany).

#### 4.1.4 Small-Angle Scattering Experiments

##### 4.1.4.1 Small-Angle X-Ray Scattering Experiments

SAXS measurements were made using a SAXSpoint 2.0 instrument by Anton Paar with point collimation (microfocus tube), equipped with a Supernova Copper radiation source (wavelength of 1.541 Å) and a 2D detector (Eiger R 1M Horizontal). This SAXS instrument can deliver structural information of dimensions between 1 and 100 nm. Small- and wide-angle data can be measured using the same sample setup at scattering angles up to 60°. In the present investigation, for LNCs of up to 74 nm (from DLS), data collection started at 0.076 nm^–1^. For a lower signal to background, the SAXS was setup under vacuum using quartz capillary sample holders. Each sample with a particle concentration of 62 mg/mL was measured for 30 min at 25 °C. A background measurement of air/buffer in the same capillary was acquired using the same settings and subtracted from the sample measurements for the data analysis.

##### 4.1.4.2 Small-Angle Neutron Scattering Experiments

SANS measurements on the LNC samples were performed at ISIS Neutron and Muon Source (Oxfordshire, UK) with a Sans2d instrument using a pulsed “white” beam, operating in a time-of-flight mode with a neutron wavelength range of 1.75–16.5 Å. The collimation of the incident beam is provided by apertures that could be selected together with the effective source distance, which is altered by inserting neutron guides to provide an appropriate beam divergence for each measurement configuration. Data were recorded on two two-dimensional detectors situated 2.4 and 4 m from the sample.

Samples with varied particle concentrations (1.24 to 6.2 mg/mL) and solvent contrasts were measured in standard quartz cells with the same parameters at temperatures of 5, 25, and 37 °C. The measured data were reduced using software provided at ISIS facility for background scattering, making allowance for the measured sample transmission, detector uniformity, and instrument noise. The data were placed on an absolute scale using the scattering from a standard sample (comprising a solid blend of protiated and perdeuterated polystyrene) in accordance with established procedures^28^ and converted to one-dimensional scattering intensity profiles *I*(*Q*) versus the momentum transfer, *Q*. Further details of the components such as detectors as well as the data reduction software can be found in the report by Heenan et al.^29^

#### 4.1.5 SAXS and SANS Data Analysis and Interpretation

SasView 5.0.3 software^30^ was used for analysis of the data obtained from SAXS and SANS experiments. Here, we also accounted for instrumental smearing effects of the SANS data. Data collected with the same LNC formulation with different solvent contrasts were simultaneously fitted to a core–shell sphere model. When analyzing the data, the SLD of the core and the solvent were kept constant at the calculated values (calculated using SLD Calculator Tool in SasView 5.0.3 software). The modeled parameters were the SLD of the shell, core radius, shell thickness, polydispersity of the radius, and the volume fraction. From the core radius and the shell thickness, the effective size of the LNCs was computed and compared with the DLS measurements. Chemical formulas and the scattering density values of the LNC components are tabulated in Table 2.

**Table 2:** Material Properties and Scattering Length Densities of LNC Components

component of LNCs	chemical formula	neutron SLD (10^–6^ Å^–2^)^b^	X-ray SLD (10^–6^ Å^–2^)^b^
Labrafac	C_8_H_16_O_2_ + C_10_H_20_O_2_	0.15	8.93
Labrafac: (dC8 + dC10) (5.89:0.31% w/v)	C_8_H_16_O_2_ + C_10_H_20_O_2_ + C_8_d_15_O_2_H + C_8_d_15_O_2_H	0.45	
Kolliphor HS 15^a^	C_20_H_40_O_4_	0.13	9.92
light water	H_2_O	–0.56	9.44
heavy water	D_2_O	6.35	

a Considering one ethylene glycol moiety.

b Calculated using SLD Calculator Tool in SasView 5.0.3 software.

## 5 Results and Discussion

### 5.1 Particle Size, Zeta Potential, and Entrapment Efficiency

LNCs were initially characterized for particle size, PDI, entrapment efficiency, and zeta potential. For SAXS experiments, LNCs with a particle concentration of 62 mg/mL were used, and for SANS experiments, LNCs were diluted to get a particle concentration in the range of 1.2–6.2 mg/mL. At a higher particle concentration of 62 mg/mL, particle radii of 35 ± 1 and 45 ± 1 nm were observed for unloaded and drug-loaded LNCs, respectively. Additionally, a PDI of 0.14 ± 0.01 was observed with drug-loaded LNCs, which is higher compared to that for unloaded LNCs (0.09 ± 0.01). Nevertheless, both the particle size and PDI of LNCs were higher at a higher particle concentration compared to LNCs with lower particle concentrations that were used for SANS measurements. LNCs at particle concentrations of 1.2–6.2 mg/mL prepared with Labrafac lipophile WL 1349 in water showed a hydrodynamic radius of 30 ± 1 nm with a PDI of 0.04 ± 0.02. For the SANS experiments, LNCs with Labrafac were prepared in deuterium oxide (D_2_O), and in a separate experiment, 5% of Labrafac was replaced with a mixture of deuterated caprylic (dC8) and deuterated capric acids (dC10). Changing the dispersion medium from water to D_2_O resulted in LNCs with a radius and PDI of 29 ± 1 nm and 0.04 ± 0.01, respectively, very similar to LNCs prepared in water. Furthermore, replacing 5% Labrafac with a mixture of dC8 and dC10 did not affect the properties of the LNCs to any greater extent, although a slight reduction in size could be seen compared to h-LNCs (Figure SI3 in the Supporting Information). The particle size and PDI values of d-LNCs were observed to be 27 ± 1 nm and 0.04 ± 0.01, respectively. These results indicate that the behavior of LNCs is little affected by inclusion of deuterium, either in the core or in the dispersion medium, making them suitable for further characterization using contrast variation and isotopic labeling for SANS experiments. In addition to unloaded LNCs, DF003-loaded LNCs were prepared in a similar manner and the formulation behavior was unaffected by the presence of deuterium. However, in all the cases where LNCs were prepared with DF003, the hydrodynamic radius was increased by about 6–8 nm compared to the unloaded LNCs. Both drug-loaded and unloaded h-LNCs and d-LNCs were measured in the concentration range of 1.2–6.2 mg/mL, and the particle size and polydispersity were unaffected in this concentration range. A detailed composition of h-LNCs and d-LNCs used for SAXS and SANS experimentation along with their characterization data can be found in Tables SI1 and SI2 in the Supporting Information.

Unloaded LNCs showed a near zero zeta potential, irrespective of the presence or absence of deuterium. However, similar to earlier results by Urimi et al.,^9^ upon loading of DF003, LNCs showed a more negative zeta potential of about −10 mV, values that were also independent of deuterium. This increase in negative zeta potential could be attributed to the strong interaction of hydrophilic DF003 with the hydrophilic headgroup region of the amphiphilic surfactant (Kolliphor HS 15). This strong interaction can be supported by the higher solubility of DF003 in Kolliphor HS 15 compared to the other components of the LNCs, as demonstrated by Urimi et al.^9^ This indicates that the drug is preferentially localized on the outer surface of the LNCs possibly in a shell-like structure formed by Kolliphor HS 15 and Phospholipon 90 H surrounding the core of LNCs, leading to a more negative surface charge. Thus, drug loading increases the size and zeta potential of LNCs. Furthermore, the entrapment efficiency of drug-loaded LNCs (both h-LNCs and d-LNCs) showed to be at >80% (detailed methodology for entrapment efficiency can be found in the Supporting Information).

### 5.2 Morphology of LNCs by Cryo-TEM

**Figure 1:**
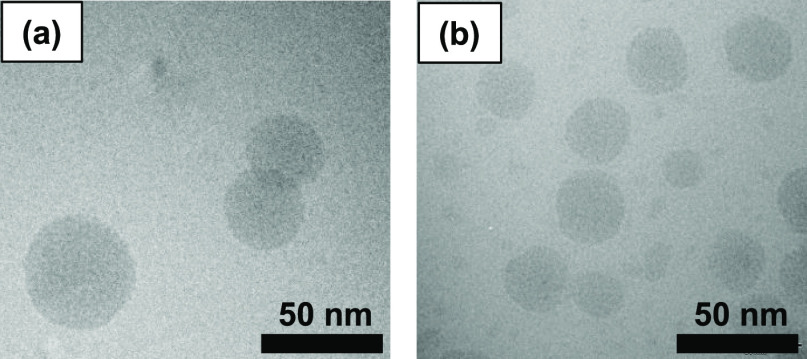
Cryogenic transmission electron micrographs of drug-loaded (a) h-LNCs and (b) d-LNCs.

To investigate the possible presence of a shell-like structure, the morphology of DF003-loaded LNCs prepared without and with a deuterated fatty acid mixture was investigated using cryo-TEM (Figure 1). The micrographs indicate the presence of spherical-shaped LNC particles. LNCs in both samples appear similar in morphology, indicating that the inclusion of d-oil for preparing d-LNCs has not affected the morphology of the resulting LNCs, as also suggested by the particle size, PDI, and zeta potential measurements. The size distribution profile of both samples is comparable to the size data measured with DLS (Figure SI3 in the Supporting Information). However, despite the presence of smaller particle population as can be seen in Figure 1, DLS showed very low PDI values (Table SI2 in the Supporting Information). This could be due to their negligible contribution to the overall scattering intensity measured with DLS. These micrographs did not reveal the internal structure of LNC particles. Due to the limited resolution of the currently used cryo-TEM technique and the poor contrast provided by polyethylene glycol, it is not possible to visualize a likely thin shell built from Kolliphor HS 15 and Phospholipon 90 H surrounding the oily core using cryo-TEM.^31^ In previous studies, it has also been demonstrated that the LNCs appear very similar with and without drug loading.^9^

### 5.3 Resolving the Structure of LNCs

#### 5.3.1 Small-Angle X-Ray Scattering

**Figure 2:**
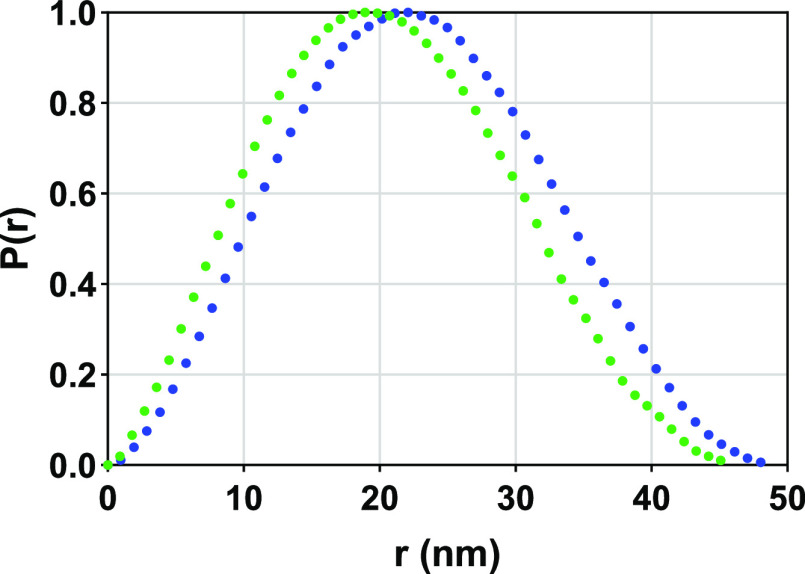
Normalized *P*(*r*) profile of unloaded (blue solid circle) and drug-loaded (green solid circle) LNCs.

Unloaded and drug-loaded LNCs were prepared with pure Labrafac in water and were measured using SAXS at a concentration of 62 mg/mL. Initially, the scattering data from both unloaded and drug-loaded LNCs were analyzed to obtain the pair distance distribution function *P*(*r*) (Figure 2). From *p*(*r*) profiles, it is clear that the particles have a spherical structure, which was also confirmed from cryo-TEM measurements (Figure 1). *P*(*r*) analysis of unloaded LNCs indicated an average radius of gyration (*R*_g_) of 17.3 ± 0.09 nm with a 49 ± 0.5 nm maximum distance between any two points in the system (*D*_max_). For the drug-loaded LNCs, the obtained *R*_g_ and *D*_max_ values were 15.8 ± 0.05 and 46 ± 0.5 nm, respectively. These *R*_g_ and *D*_max_ values indicate that the LNCs with the drug are slightly smaller in size compared to unloaded LNCs.

**Figure 3:**
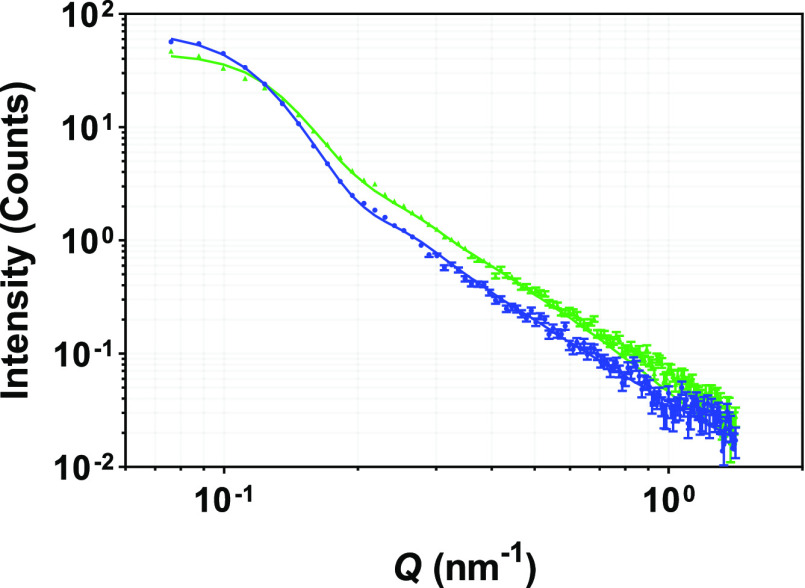
SAXS data from unloaded (blue solid circle) and drug-loaded (green solid circle) LNCs fitted with a core–shell sphere model. Solid lines represent the best fits to the experimental scattering data. Error bars are almost within the size of the symbols for most of the scattering data at low *Q* values.

Based on these observations, a sphere model and a core–shell sphere model^32^ were fitted to the scattering data to get the structural details of the LNC particles. Scattering data along with the best model fits are presented in Figure 3. It can be seen from Figure SI4 in the Supporting Information that the scattering data did not fit well with the sphere model, suggesting the presence of additional structural features. Significant improvement was observed when a shell was added to the sphere model (Figure 3). Structural parameters from the data fitting of the LNCs are tabulated in Table 3. Furthermore, it is evident from the scattering profiles shown in Figure 3 that the structures of unloaded and drug-loaded LNCs are similar and were well fitted with a core–shell sphere model, but they differ in size. From the best data fits, the average core radius and shell thickness of unloaded LNCs were found to be 21.7 ± 0.2 and 2.6 ± 0.1 nm, respectively. Upon drug loading, the average core radius was reduced to 18.5 ± 0.2 nm, but the shell thickness was increased to 3.6 ± 0.1 nm. Taken together, this means that drug loading into LNCs led to a decrease in the average overall size of core–shell LNC particles. These size parameters obtained from modeling SAXS data correlate well with the *P*(*r*) analysis (Figure 2) but appear to go against the results from DLS presented above where the particles appeared larger in response to the drug being present (Table SI2). DLS measures the diffusion coefficient, and binding the drug molecule on the surface of LNCs can potentially change the diffusion coefficient. SAXS is more accurate in determining the size of these LNC particles, as it measures the particle size directly.

**Table 3:** Structural Information about the LNCs as Determined by SAXS, SANS, and DLS Techniques

parameter	unloaded LNCs	DF003-loaded LNCs
*P*(*r*) analysis of small-angle X-ray scattering (SAXS) data		
*R*_g_ (nm)	17.3 ± 0.09	15.8 ± 0.05
*D*_max_ (nm)	49 ± 0.5	46 ± 0.5
shape model analysis of small-angle X-ray scattering (SAXS) data		
core radius (nm)	21.7 ± 0.2	18.5 ± 0.2
shell thickness (nm)	2.6 ± 0.1	3.6 ± 0.1
total radius (nm)	24.3 ± 0.3	22.1 ± 0.3
volume fraction^a^	0.092	0.092
polydispersity	0.20	0.35
χ^2^	2.4	3.7
small-angle neutron scattering (SANS)		
core radius (nm)	20.0 ± 0.9	20.2 ± 0.6
shell thickness (nm)	≤1.5	∼2 ± 0.5
total radius (nm)	21.5 ± 0.9	22.2 ± 1.1
shell hydration (%)	50	70
volume fraction^b^	0.009	0.009
polydispersity	0.20	0.25
SLD of shell	3.3	4.5
dynamic light scattering (DLS)		
hydrodynamic radius (nm)	30.0 ± 1.0	36.0 ± 1.0
polydispersity	0.04 ± 0.02	0.07 ± 0.01
zeta potential (mV)	–3.7 ± 1.6	–13.6 ± 0.8

a Volume fractions estimated using a core–shell sphere model and the values are in close approximation to a theoretical volume fraction of 0.062.

b Volume fractions estimated using a core–shell sphere model and the values are in close approximation to a theoretical volume fraction of 0.0062.

With SAXS, we measure the average particle size; thus, in conclusion, the decrease in the average size of the LNC particle upon drug loading could be explained by a shift in particle size distribution to smaller dimensions, resulting in a smaller average size. Despite the average decrease in size, the shell is larger in drug-loaded LNCs compared to the unloaded ones, indicating the presence of the drug on the surface of the LNC particles as measured by SAXS.

#### 5.3.2 Small-Angle Neutron Scattering

**Figure 4:**
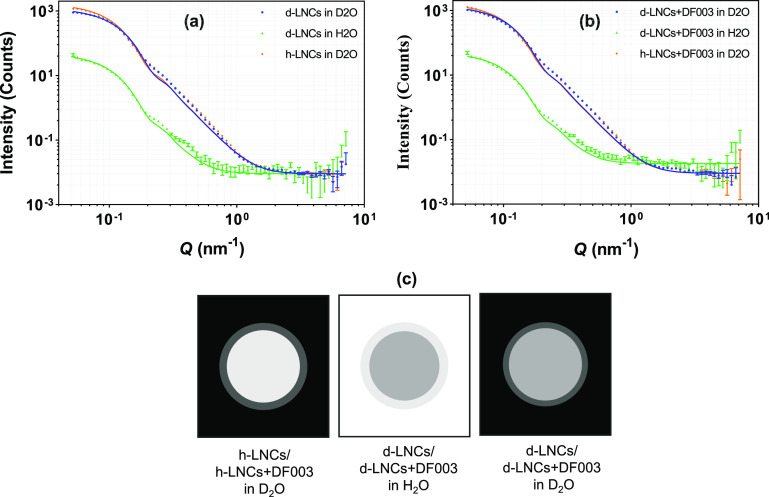
SANS scattering data from (a) unloaded LNCs and (b) DF003-loaded LNCs prepared with varied contrast in the core and in the dispersion medium. Solid lines represent the best fits to the experimental scattering data. (c) Schematic depiction of varied contrasts in the core and in the dispersion medium. Error bars are almost within the size of the symbols for most of the scattering data.

Building on the SAXS data of LNCs, the SANS data were fitted to a core–shell sphere model. Figure 4 displays the scattering intensity curves along with a schematic sketch of contrast differences for LNCs with varying contrasts.

##### 5.3.2.1 Resolving the Core

To investigate the structural details of the nanoparticles, LNCs were prepared with pure hydrogenous oil (i.e., Labrafac) and Labrafac mixed with a 5% mixture of dC8 and dC10 oils to increase the contrast in the core for neutrons. Along with this, the dispersion medium was changed from H_2_O to D_2_O to highlight different structural aspects of LNCs. In Figure 4a, the scattering data from unloaded h-LNCs and d-LNCs prepared in H_2_O and D_2_O are displayed, where most of the signal comes from the core. The best fits from a core–shell sphere model were obtained with a core radius of 20.0 ± 0.9 nm. The fits slightly deviate from the scattering data between *Q* = 0.2–0.8 nm^–1^. The model was kept simple to get the best possible and relevant structural information of the LNCs. A schematic picture with contrast variation in the core of LNCs and the dispersion medium is shown in Figure 4c.

##### 5.3.2.2 Resolving the Shell

SANS data fitting with a core–shell sphere model demonstrated the presence of a highly hydrated shell surrounding the core of LNCs. In the case of unloaded LNCs, the shell hydration was found to be of the order 50% and this was seen by an increase in SLD from a calculated value of 0.13 to 3.3 from the data fitting. Based on the data fitting, the shell thickness was found to be <1.5 nm; however, this diffuse shell is difficult to model precisely with respect to thickness, as the signal is weak. The high hydration of the shell was further confirmed by experiments where the signal of the core was matched out by the dispersion medium and the remaining very weak signal only came from the shell (Figure SI5 in the Supporting Information). It is clear that the hydration of the shell of these soft particles makes it difficult to model their exact thickness. Previously, it has been found that Kolliphor HS 15 orients toward the aqueous phase and does constitute a sufficiently rigid shell,^11^ and data represented herein support this speculation.

Taking together, the information obtained with SAXS and SANS suggests that the LNC particles have a core–shell structure. The shell is assumed to be made up of a combination of the surface active agents Kolliphor HS 15 and Phospholipon 90 H, as they are associated together at the interface of the particle and the surrounding medium. The predominant thickness of the shell results from the PEG chains of Kolliphor HS 15. Data from SAXS and SANS experiments provide an indication on the extent of the contribution of shell thickness to the overall size of the LNCs. However, owing to the dynamic and interfacial nature of the shell components and polydispersity associated with the actual particles, it is highly challenging to precisely measure the thickness of the shell even with the advanced characterization tools. Polydispersity values of 0.2 and 0.25 for the unloaded and drug-loaded LNCs, respectively, were obtained through modeling the SANS data. A similar increase in the polydispersity of LNCs with drug loading was observed with DLS measurements (Table SI2) and cryo-TEM observations.

##### 5.3.2.3 Addition of the Drug

**Figure 5:**
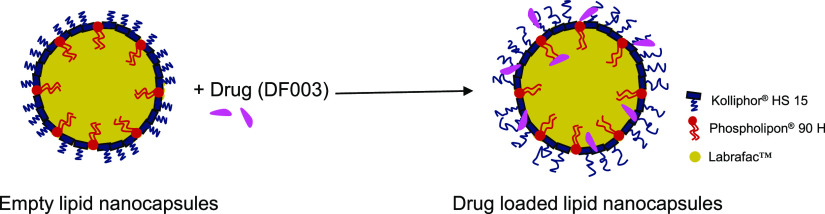
Proposed structure of empty and DF003-loaded lipid nanocapsules. Drug-loaded LNCs are proposed to have a higher disorder in the way the surfactants are packed around the oily core by the presence of the drug.

Drug-loaded h-LNCs and d-LNCs were measured with SANS in the same way as unloaded LNCs (Figure 4b). The scattering intensity profiles followed a similar pattern and a core–shell sphere model fits well, similar to the unloaded LNCs (Figure 4a vs Figure 4b), indicating a retained overall core–shell structure upon drug loading. The drug loading resulted in a higher scattering intensity relative to unloaded LNCs, indicating an increased overall size with drug loading. The best possible fit showed a core radius of 20.2 ± 0.6 nm for drug-loaded LNCs, which is very similar to 20.0 ± 0.9 nm obtained for unloaded LNCs (Table 3). Additionally, similar to unloaded LNCs, the shell in drug-loaded LNCs is highly hydrated; however, this hydration is even higher at ∼70%, compared to the shell hydration of unloaded LNCs (50%). Further data analysis showed a shell thickness of ∼2 ± 0.5 nm, which is thicker compared to the shell thickness observed for unloaded LNCs. This increased shell hydration and thickness can be explained by the hydrophilic nature of DF003 localizing it on the surface of the particles (as confirmed by particles becoming more negatively charged with drug loading, Table SI2). DF003 may form hydrogen bonds with water, making the shell more hydrated compared to unloaded LNCs. Additionally, drug localization and surface charge may imply a different positioning of the surfactant monomers surrounding the core of LNCs. These combined effects may lead to an increase in the shell thickness. A schematic illustration of empty and drug-loaded LNCs is shown in Figure 5.

##### 5.3.2.4 Effect of Concentration

**Figure 6:**
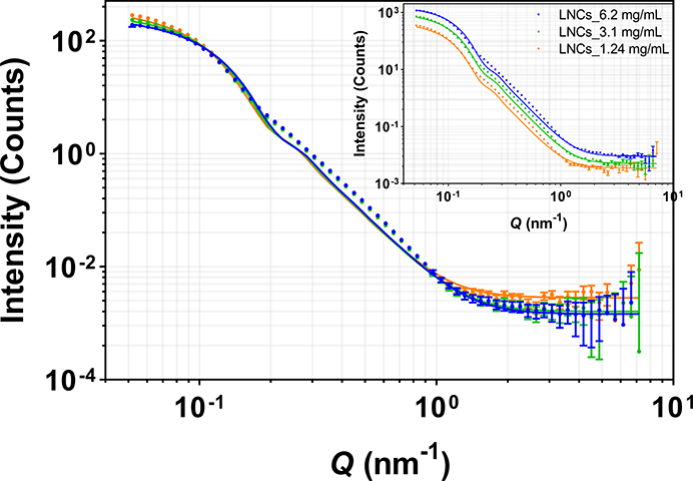
Effect of particle concentration on SANS scattering data from unloaded h-LNCs prepared in D_2_O. Data were normalized with respect to concentration, and the inset shows non-normalized SANS data. Error bars are almost within the size of the symbols for most of the scattering data.

**Figure 7:**
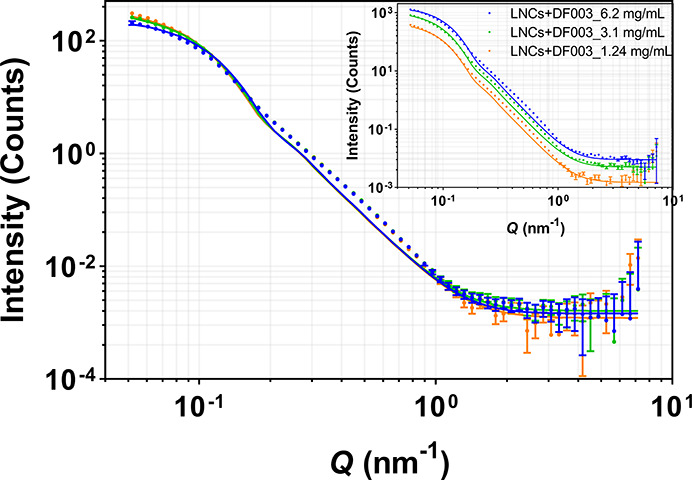
Effect of particle concentration on SANS scattering data from DF003-loaded h-LNCs prepared in D_2_O. Data were normalized with respect to concentration, and the inset shows non-normalized SANS data. Error bars are almost within the size of the symbols for most of the scattering data.

Drug-loaded and unloaded LNCs with or without a deuterated core were prepared using H_2_O or D_2_O as the dispersant and were measured with SANS at different particle concentrations, 1.24, 3.1, and 6.2 mg/mL. Figures 6 and 7 represent the scattering intensity profile and the model fit from unloaded and drug-loaded h-LNCs prepared in D_2_O. The data indicate that the overall structure is retained upon dilution (for both unloaded and drug-loaded LNCs). The signal intensities vary according to volume fractions of LNCs. The average core radii for unloaded and drug-loaded LNCs at different concentrations were observed to be 20.9 ± 1.0 and 20.7 ± 1.0 nm, respectively, indicating that the core remains unchanged in size with particle concentration irrespective of the presence or absence of the drug.

##### 5.3.2.5 Effect of Temperature

**Figure 8:**
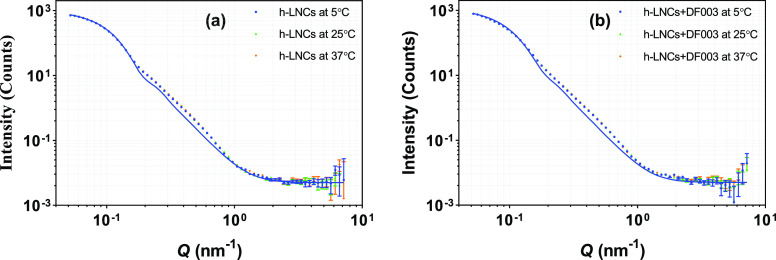
Effect of temperature (5, 25, and 37 °C) on SANS scattering data from (a) unloaded h-LNCs and (b) DF003-loaded h-LNCs prepared in D_2_O. Error bars are almost within the size of the symbols for most of the scattering data.

SANS measurements on h-LNCs and d-LNCs (with and without the drug) were carried out at 5, 25, and 37 °C to investigate the impact of temperature on the internal structure of LNCs. The scattering intensity profile with the best fit from nondeuterated LNCs prepared in D_2_O is given in Figure 8. The samples were diluted 20 times with D_2_O before the measurement. It can be noted that the scattering intensity profiles generated at different temperatures are very similar to each other, indicating that the structure of these LNCs is stable and that the measurements are unaffected over this temperature range. All different temperatures showed the best fit with a core diameter of 21.5 nm.

##### 5.3.2.6 Effect of Salt

**Figure 9:**
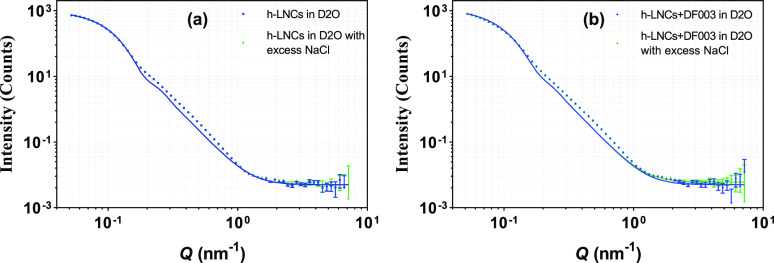
Effect of excess salt on SANS scattering data from (a) unloaded h-LNCs and (b) DF003-loaded h-LNCs prepared in D_2_O. Error bars are almost within the size of the symbols for most of the scattering data.

In our previous work, we have demonstrated the stability of DF003-loaded LNCs with hyaluronic acid sodium to check for the suitability of LNCs for ocular administration.^9^ To further investigate their stability, both unloaded and drug-loaded h-LNCs were diluted with sodium chloride dissolved in D_2_O to a final salt concentration of ∼5 mg/mL and SANS profiles were generated (Figure 9). Scattering profiles remained the same with and without the presence of excess NaCl. This indicates that excess salt did not change the overall core–shell structure for neither unloaded nor drug-loaded LNCs. SANS data along with size data measured with DLS (Table SI2) show that the LNCs are stable and nonaggregating without any changes in the internal structure at the studied salt concentration.

## 6 Conclusions

Structural aspects of LNCs were studied using a combination of different characterization techniques, including DLS, cryo-TEM, SAXS, and SANS. Information about the overall particle size and morphology was obtained from DLS and cryo-TEM, whereas SAXS and SANS provided more detailed information about the internal structure of LNCs. The results indicate the presence of a core–shell structure, which is retained after drug loading into LNCs. Modeling of both SAXS and SANS data showed an increase in the thickness of the shell for drug-loaded LNCs, compared to unloaded LNCs. It could be further observed from SANS data that drug loading of LNCs resulted in higher hydration of the shell, leading to the increase in shell thickness. The hydrophilic nature of DF003 correlates well with the increased hydration of the shell and strengthens the conclusion of a preferential localization of DF003 to the shell. Moreover, the concluded localization of DF003 to the shell was further confirmed by a negative surface potential of drug-loaded LNCs, compared to a near neutral surface potential of unloaded LNCs. The LNC core–shell configuration was found to be unaffected by particle concentration, temperature, or the presence of excess salt, demonstrating the stability of these particles. However, for drug-loaded LNCs at higher LNC particle concentrations (62 mg/mL) as used for SAXS measurements, we found that the average LNC particle size decreased, indicating a shift in the size distribution of the core–shell particles to smaller sizes. Further investigation into the effect of LNC assembly with the drug present at different particle concentrations could shed more light onto what causes this shift in dynamics as seen with the SAXS measurements. The findings from this work can help in better understanding of the physicochemical properties of LNCs and the effect of drug loading. Overall, this complementary approach can be implemented in finding out the structural details of various other novel drug delivery systems.

## Supporting Information Available

Chemical structures of the drug (DF003), Kolliphor HS 15, additional experimental methods, DLS and encapsulation efficiency results of LNC samples, SAXS model fitting with a sphere model, and SANS data in contrast match conditions (PDF)
