# Supplementary material for: Structural Characterization Study of a Lipid Nanocapsule Formulation Intended for Drug Delivery Applications Using Small-Angle Scattering Techniques
Source: Mol Pharm. 2022 Feb 28;19(4):1068–77. doi: 10.1021/acs.molpharmaceut.1c00648 (PMC8985204; doi:10.1021/acs.molpharmaceut.1c00648)
Supplement: Supplementary file 1 — mp1c00648_si_001.pdf [file mp1c00648_si_001.pdf]

Supporting information to

Structural characterization study of a lipid nanocapsule formulation intended for drug delivery applications using small angle scattering techniques

Dileep Urimi [†‡*], *Maja Hellsing*[†], *Najet Mahmoudi*[§], *Christopher Söderberg*[†], *Ronja Widenbring*[†], *Lars Gedda*[††], *Katarina Edwards*[††], *Thorsteinn Loftsson*[‡], *Nicolaas Schipper*[†]

[†]RISE Research Institutes of Sweden, Division Bioeconomy and Health, Chemical Process and Pharmaceutical Development, Forskargatan 18, 151 36 Södertälje, Sweden

[‡]Faculty of Pharmaceutical Sciences, School of Health Sciences, University of Iceland, Hofsvallagata 53, IS-107 Reykjavík, Iceland

[§]ISIS Pulsed Neutron and Muon Source, Rutherford Appleton Laboratory, Didcot OX11 0QX, UK.

[††]Department of Chemistry – Ångström laboratory, Uppsala University, Box 573, Uppsala, SE-751 23, Sweden

*Corresponding author: Dileep Urimi

E-mail: dileep.urimi@ri.se

Tel.: +46-703486575

Chemical structures

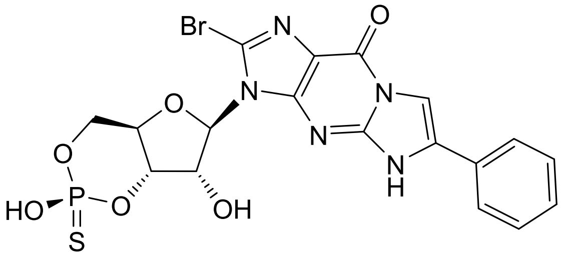

Figure SI1. Molecular structure of drug candidate, DF003

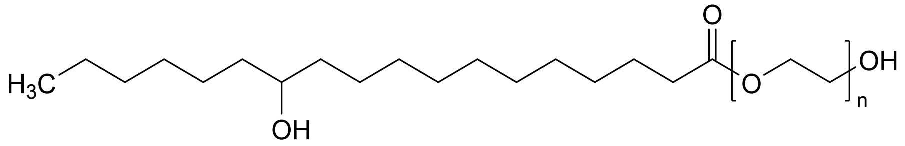

Figure SI2. Molecular structure of Kolliphor® HS 15

Preparation of lipid nanocapsules

Details of h-LNCs and d-LNCs prepared for SANS experiments are mentioned in Table SI1.

Table SI1. Details of the formulations used for structural characterization.

Formulation	Proportion of Labrafac™ (% w/v)	Proportion of dC8+dC10 (% w/v)	Dispersion medium	Drug loading (drug: total oil)
For SAXS experiments (particle concentration (62 mg/mL)				
F1	6.20	-	H_2O	-
F2	6.20	-	H_2O	1:32
For SANS experiments (Particle concentration (1.2-6.2 mg/mL)				
F3	6.20	-	H_2O	-
F4	6.20	-	D_2O	-
F5	5.89	0.31	D_2O	-
F6	6.20	-	H_2O	1:32
F7	6.20	-	D_2O	1:32
F8	5.89	0.31	D_2O	1:32
F9*	6.20	-	D_2O	-
F10*	6.20	-	D_2O	1:32

*Final NaCl concentration of 5 mg/mL for SANS measurements

Determination of encapsulation efficiency

Entrapment efficiency (% EE) and drug loading of DF003 into LNCs were measured with indirect method. Initially, soluble unentrapped drug was separated from the formulations by centrifuging 0.5 mL of drug loaded formulations at 10000x g for 5 minutes. Centrifugation was performed in individual Amicon® Ultra 0.5 centrifugal units with 50 kD membrane. After 5 min, clear filtrate without LNCs was collected and DF003 was quantified using UPLC-UV. The % EE was calculated using equations (2).

$$\% \text{ EE } = \frac{\text{Amount total DF003 - Amount of unentrapped DF003}}{\text{Amount total DF003}} \times 100 \qquad (1)$$

Characterization data of LNCs

Table SI2. Physicochemical characterization of LNCs used for the structural characterization.

**Formulation**	**Particle radius (nm)**	**Polydispersity Index (PdI)**	**Zeta potential (mV)**	**Entrapment efficiency (%)**
		For SAXS experiments (particle concentration (62 mg/mL)		
F1	35 ± 1	0.09 ± 0.01	-	-
F2	45 ± 1	0.14 ± 0.01	-	86
		For SANS experiments (Particle concentration (1.2-6.2 mg/mL)		
F3	30 ± 1	0.04 ± 0.02	-3.7 ± 1.6	-
F4	29 ± 1	0.04 ± 0.01	-1.3 ± 0.3	-
F5	27 ± 1	0.04 ± 0.01	-3.7 ± 2.1	-
F6	36 ± 1	0.07 ± 0.01	-13.6 ± 0.8	86
F7	37 ± 1	0.05 ± 0.01	-13.1 ± 0.2	93
F8	33 ± 1	0.04 ± 0.01	-9.6 ± 0.3	87
F9*	30 ± 1	0.03 ± 0.00	-2.0 ± 0.4	-
F10*	36 ± 1	0.07 ± 0.01	-4.5 ± 1.0	-

*Final NaCl concentration of 5 mg/mL for SANS measurements

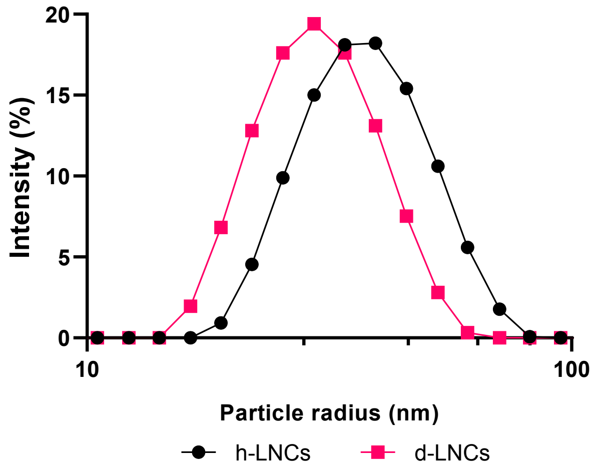

Figure SI3. Comparison of size distribution profiles of h-LNCs and d-LNCs obtained with dynamic light scattering technique. Samples were at a concentration of 6.2 mg/mL. Particle size in this graph refers to the intensity weighted mean hydrodynamic radius.

<u>Small angle X-ray scattering (SAXS)</u>

SAXS data of h-LNCs when fitted with a sphere model indicating a poor fit (Figure SI4) and thus emphasizing the presence of an internal structure in the LNC particles.

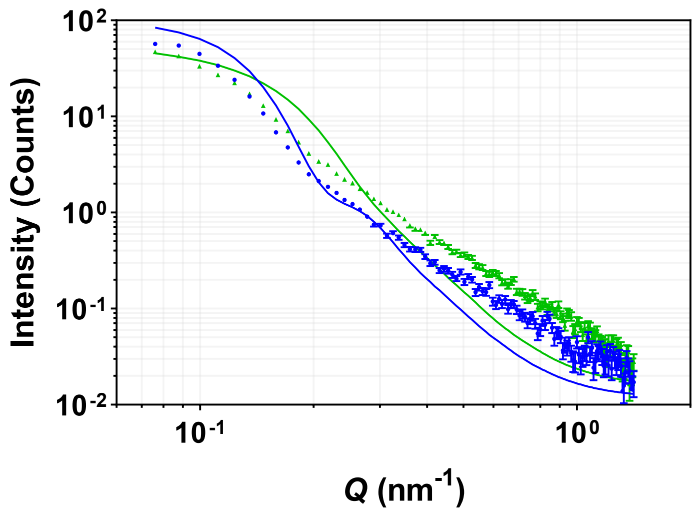

Figure SI4. SAXS data from unloaded (●) and drug-loaded (●) LNCs fitted with a sphere model. Solid lines represent the best fits to the experimental scattering data. Error bars are almost within the size of the symbols for most of the scattering data.

Small angle neutron scattering (SANS)

SANS data was generated on d-LNCs in a mixture of H_2O and D_2O to match out the contrast from the core with the contrast from the dispersion medium to be able to get a reasonable signal from the shell (Figure SI5). However, as can be seen in Figure SI5, the signal is very weak and moreover the shell is much more hydrated which made it difficult to estimate the accurate thickness of the shell.

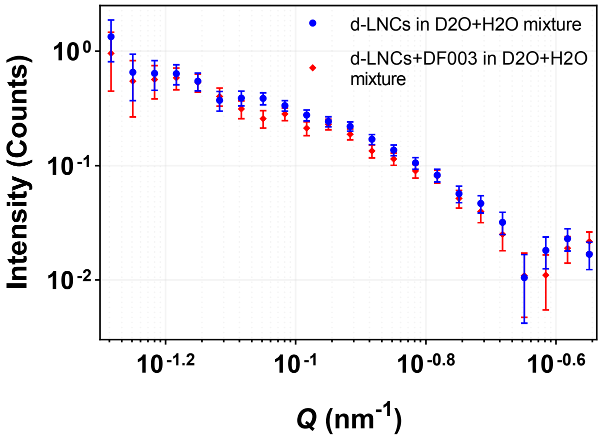

Figure SI5. SANS data from d-LNCs with and without drug loading prepared in a mixture of H_2O and D_2O for matching out the core with the solvent.
